# Metagenomic and metabolomic analyses reveal differences in rumen microbiota between grass- and grain-fed Sanhe heifers

**DOI:** 10.3389/fmicb.2024.1336278

**Published:** 2024-05-13

**Authors:** Xinyu Zhang, Wei Wang, Yajing Wang, Zhijun Cao, Hongjian Yang, Shengli Li

**Affiliations:** State Key Laboratory of Animal Nutrition, Beijing Engineering Technology Research Center of Raw Milk Quality and Safety Control, College of Animal Science and Technology, China Agricultural University, Beijing, China

**Keywords:** metabolites, microbiota, grass-fed, grain-fed, Sanhe heifers

## Abstract

**Introduction:**

The aim of this study was to investigate the effects of diets on the composition and function of rumen microbiome and metabolites in Sanhe heifers.

**Methods:**

Metagenomic and metabolomic analyses were performed using rumen fluid samples collected from Sanhe heifers (*n* = 20) with similar body weights and ages from grass-fed and grain-fed systems.

**Results:**

The grain-fed group exhibited more intensive rumen fermentation than the grass-fed group. However, the grass-fed group exhibited carbohydrate metabolism and methane production higher than that of the grain-fed group; these increases were observed as a higher abundance of various bacterial phyla (Firmicutes, Bacteroidetes, Actinobacteria, Lentisphaerae, and Verrucomicrobia), families (Lachnospiraceae, Eubacteriaceae, and Eggerthellaceae), and the archaeal family Methanobacteriaceae. A comparison of genes encoding carbohydrate-active enzymes, using Kyoto Encyclopedia of Genes and Genome profiles, revealed noteworthy differences in the functions of rumen microbiota; these differences were largely dependent on the feeding system.

**Conclusion:**

These results could help manipulate and regulate feed efficiency in Sanhe cattle.

## 1 Introduction

Sanhe cattle, a dual-purpose breed, are famous for their excellent milk (i.e., high in fat) and high meat yields (Xu et al., 2017). Sanhe cattle have rough feeding tolerance (Hu et al., 2019), strong disease resistance (Usman et al., 2017), and stable genetic performance. In previous reports, we introduced Sanhe cattle to an altitude of 3,650 m in Tibet and observed improved nutrient digestibility, indicating that Sanhe cattle exhibit strong adaptability to high-altitude environments (Zhang X. et al., 2022). However, the effects of feeding regimens on the composition and functions of gut microbiota in Sanhe heifers have not been explored.

Ruminant-derived products are important sources of meat and milk, providing high-quality protein for human consumption. Beyond being an organ, the rumen can be characterized as a complex microbial ecosystem that hosts numerous bacteria, fungi, archaea, and protozoa (Mackie and White, 1990; Russell and Rychlik, 2001); the microbial communities hosted in the rumen shape the amount of energy ruminants can extract from their food, thereby directly impacting agricultural productivity. In an anaerobic environment, the rumen microbiota digest (through fermentation) the feed consumed by cattle and produce volatile fatty acids (VFAs). VFAs, including acetate, propionate, butyrate, and valerate, affect carcass yield and meat quality traits in beef cattle. The component microbes in the rumen microbiota work together to maintain the stability of the rumen environment, associated with rumen fermentation (Morgavi et al., 2013), feed efficiency (Jewell et al., 2015; Shabat et al., 2016), and methane emissions. Various factors, including diet, age (Jami et al., 2013; Guo et al., 2020), antibiotics (Yousif et al., 2018; Holman et al., 2019), and environment, can affect the microbiota of ruminants. However, among these factors, diet has the greatest impact on the microbiota of the digestive tract. For example, cows fed a high-fiber diet expressed a higher relative abundance of *Fibrobacterota* in the rumen, whereas those fed a high-concentrate diet had a higher relative abundance of Bacteroidetes (Fernando et al., 2010). Similarly, increased dietary proportions of grain and starch lower bacterial richness and diversity in the rumen of cattle (Mao et al., 2013; Petri et al., 2013). Moreover, feeding regimes, such as grass- and grain-feeding, have been shown to affect the physiological health of cows, and the organoleptic and nutritional quality of the resultant beef and milk.

Metagenomics and metabolomics based on DNA levels and metabolic differences have gained increased research interest as effective tools for understanding the functional potential of the rumen microbiome (Li et al., 2018). A few studies have also used combined meta-omics analyses to explore rumen microbiome functions. Recently, two studies have explored the effects of grass- and grain-fed diets on the rumen microbiota of sheep and yaks using 16S rDNA (Belanche et al., 2019; Xu et al., 2021). We hypothesized that changes in the rumen microbiota of Sanhe heifers caused by changes in the diet might explain the changes in VFA and metabolites in the two different feeding systems.

In this study, using metagenomic and metabolomic analyses, we investigated the effect of various feeding regimens on the composition of rumen microbiota and metabolites of Sanhe heifers. These efforts are meant to bring forth insights regarding the role of the rumen microbiota in digestion and metabolism and can help assist in manipulating and regulating feeding regimens to realize maximum feed efficiency.

## 2 Materials and methods

### 2.1 Experimental design

The Sanhe heifers utilized in our study were sourced from the Inner Mongolia Autonomous Region (119°57′E, 47°17′N; 700 m altitude). In May 2020, 150 Sanhe heifers of similar age (12 months) and body weight were chosen from a herd of over 500 Sanhe cattle that shared the same father and were housed at the Xieertala farm in Inner Mongolia. These heifers were subsequently divided into two groups, each comprising 75 individuals. One group remained at Xieertala farm (grain-fed group), while the other was transported to nearby pastures for grass feeding (grass-fed group). The grain-fed Sanhe heifers were provided with a total mixed ration (TMR) twice daily at 06:30 a.m., and 17:30 p.m. The TMR was formulated based on the National Research Council guidelines, aiming to meet the nutrient requirements of Sanhe heifers under the given conditions (NRC, 2001). The grain-fed group fed with the diet with dry matter 50.79%, organic matter 90.45%, crude protein 15.70%, ether extract 2.92%, neutral detergent 38.98%, acid detergent fiber 22.36%. The TMR was sampled weekly, dried, ground, and subjected to chemical analysis. Water was provided *ad libitum*. The grass-fed group had *ad libitum* access to pasture.

Ten healthy Sanhe heifers with similar ages (14–15 months) and mean body weights (340.71–328.65 kg) were randomly selected from each group. Rumen fluid samples were collected at 06:30 a.m. before morning feeding. The samples were divided, with one part immediately frozen in liquid nitrogen (−196°C) for bacterial analysis, and the other stored at −20°C for examining rumen fermentation parameters.

This experiment received approval from the Ethical Committee of China Agricultural University (project number AW22121202-1-2).

### 2.2 Rumen fermentation parameter analysis

To assess the VFA concentrations in rumen fluid samples, each sample was thawed and subjected to centrifugation. The quantification of the supernatant was conducted, following a protocol outlined in a prior study by Erwin et al. (1961).

### 2.3 The process of metagenome sequencing

DNA was extracted from samples using a kit. The quantity and quality of DNA were assessed through TBS-380 and NanoDrop2000 instruments. A 1% agarose gel was used to confirm DNA integrity. Subsequently, DNA extract was fragmented to an average size of about 400 bp using Covaris M220 (Gene Company Limited, China) for paired-end library construction. Paired-end libraries were determined through the NEXTFLEX Rapid DNA-Seq kit. Adapters were attached to DNA fragments. Sequencing was detected using an Illumina NovaSeq machine.

After obtaining paired-end Illumina reads, the “fastp” tool (Chen et al., 2018) removed adapters and excluded reads (length ≤ 50 bp, quality value ≤20, or containing N bases). Metagenomic data compilation involved generating condensed de Bruijn plots using MEGAHIT (Li et al., 2015 non-redundant database, version 1.1.2). Synonyms (length ≥ 300 bp) were chosen to ensure data integrity. These synonyms were used for downstream genetic analysis and annotation.

Open reading frames (ORFs) within the compiled isoforms underwent prediction through Prodigal (Hyatt et al., 2010). Application of NCBI translation tables facilitated the identification of predicted ORFs with a length of ≤100 bp, subsequently translating them into amino acid sequences. The establishment of a non-redundant gene catalog was accomplished employing CD-HIT (Fu et al., 2012). High-quality reads were aligned to the catalog of non-redundant genes using SOAPaligner (Li et al., 2008), and gene abundance was computed with a 95% identity threshold. For taxonomic annotation, Diamond (Buchfink et al., 2015) aligned representative sequences from the non-redundant gene catalog to the non-redundant database, applying an e-value limit of 1e-5. KEGG annotation was executed through Diamond’s analysis against the KEGG database, maintaining an e-value limit of 1e-5. Carbohydrate active enzyme (CAZyme) annotations were conducted using HMMER hmmscan against the CAZy database, implementing an e-value cutoff of 1e-5.

### 2.4 Liquid chromatography-mass spectrometry analysis

Metabolite extraction from rumen fluid samples involved mixing with a cold solvent mixture. After vortexing and ice bath treatment, samples were centrifuged, and the supernatant was determined by liquid chromatography-mass spectrometry (LC–MS).

Rumen fluid metabolites underwent separation employing an ultra-high-performance liquid chromatography system (1,290 Infinity LC; Agilent Technologies, Santa Clara, CA, United States) coupled with a quadrupole time-of-flight mass spectrometer (TripleTOF 6,600, AB Sciex, Framingham, MA, United States). The analysis utilized a 2.1 mm × 100 mm ACQUIY UPLC BEH 1.7 μm column (Waters, Milford, MA, United States). The mobile phases facilitated positive and negative ion electrospray ionization mode. Gradient conditions were meticulously set, beginning at 85% B for 1 min, linearly decreasing to 65% over 11 min, followed by a linear decrease to 40% for 0.1 min, maintaining at 40% for 4 min, and concluding with an increase to 85% over 0.1 min. The system underwent reequilibration for 5 min before each subsequent run. The electrospray ionization source operated under specified conditions: ion source gas 1 at 60, gas 2 at 60, curtain gas at 30, source temperature at 600°C, and ionization spray voltage fluctuating ±5,500 V. During mass spectrometry acquisition, the instrument’s m/z range spanned from 60 to 1,000 Da, with a build-up time of the time-of-flight mass spectrometry scans set at 0.20 s/segment. Signal acquisition occurred in Auto-MS/MS mode within the m/z range of 25–1,000 Da, and the production scan’s accumulation time was established at 0.05 s/segment. Utilizing information-dependent acquisition, production scans operated in high-sensitivity mode with a fixed collision energy of 35 V ± 15 eV and a de-clustering potential of ±60 V.

Raw MS data in wiff.scan files were converted to MzXML files via ProteoWizard MSConvert, and subsequent processing included feature detection, retention time correction, and result alignment using XCMS. Metabolite identification hinged on precise mass spectrometry analysis (precision <25 ppm) and MS/MS data corresponding to standard databases. For extracted ion characterization, variables with non-zero values >50% were retained in at least one group. Multivariate statistical analyses, including Pareto scaling, principal component analysis (PCA), and partial least squares discriminant analysis (OPLS-DA), were executed through the MetaboAnalyst web system. Model robustness underwent assessment via leave-one-out cross-validation and response replacement tests. Differences in metabolites between the low and high abundance groups were determined based on a combination of statistically significant variables’ effect on prediction (VIP) values in the OPLS-DA model and two-tailed Student’s t-tests (*p*-values) on raw data. Significance criteria encompassed VI*p* values greater than 1.0 or less than 0.05, and P values less than 0.05. Metabolite identification drew upon three databases, namely, KEGG, Human Metabolome, and Bovine Metabolome. Enrichment of KEGG metabolic pathways by distinct metabolites was evaluated using the KEGG database, and pathway significance was ascertained through Fisher’s exact test.

### 2.5 Analysis of metagenomes and metabolomes

The t-test in SPSS software (version 22.0, SPSS, Inc., Chicago, IL, United States) was used to compare the values of rumen fermentation parameters between the two groups. Only microbial taxa with relative abundance greater than 0.01% were included in this study. Compositional profiles of the rumen microbiota were summarized at the level of phylum, family, genus, and species, whereas the archaeal community was summarized at the level of species. We analyzed the α-diversity index using the Wilcoxon rank test. Principal Coordinate Analysis (PCoA) was performed in R based on the Bray-Curtis heterogeneity matrix, and the results were visualized using the “ggplot2” package. To compare the relative abundance and microbiota function of the two groups of organisms at the phylum, family, genus, and species levels, we used the Wilcoxon method in the R software (version 4.0.5). In addition, we used the “corrplot” package (author: Taiyun Wei; publication date: 2017; version: 0.84) in the “Psych” software package (author: W Revelle; publication date: 2016; version: 1.6.9) to perform Spearman rank correlation analysis. All data are reported as means, and *p* < 0.05 was considered significant.

## 3 Results

### 3.1 Shifts in fermentation parameters of rumen microbiota of Sanhe heifers

Table 1 presents the rumen fermentation parameters in Sanhe heifers under different feeding systems. The contents of acetate, butyrate, isovalerate, and total VFAs were significantly higher in the grass-fed group than those in the grain-fed group (*p* < 0.05). Moreover, the propionic acid content of the grain-fed group was significantly higher than that of the grass-fed group (*p* = 0.06).

**Table 1:** Parameters of rumen fluid fermentation in two groups of Sanhe heifers.

Parameter	Group^a^		SEM	*p-*value
	Grass-fed	Grain-fed		
Acetate (mmol/L)	34.24	45.75	9.55	0.01
Propionate (mmol/L)	20.13	25.85	5.97	0.06
Butyrate (mmol/L)	14.68	20.41	5.14	0.03
Isovalerate (mmol/L)	2.36	2.99	0.55	0.02
TVFAs (mmol/L)	73.27	97.98	21.59	0.02

a Grass-fed and Grain-fed groups; TVFAs, total volatile fatty acids; SEM, standard error of the mean.

### 3.2 Effects of feeding regimens on the composition of rumen microbiota communities in Sanhe heifers

#### 3.2.1 Profiling of the rumen metagenome

Metagenome sequencing obtained 2,349,913,544 reads, with 117,495,677 ± 11,409,254 reads (mean ± standard error of the mean [SEM]) per sample (Supplementary Table S1). After quality control and removal of host genes, we obtained 2,319,213,390 ± 2,531,029 reads. Subsequently, *de novo* assembly yielded 36,567,343 contigs (N50 length was 609 ± 11 bp) with 1,828,367 ± 65,016 per sample.

The metagenome of Sanhe heifers identified five domains comprising 14 kingdoms; 245 phyla; 490 classes; 1,031 orders; 2,130 families; 6,159 genera; and 37,528 species. The metagenome comprised 86.83% bacteria, 8.44% Archaea, 3.53% eukaryotes, and 1.11% viruses (Supplementary Figure S1A). At the species level, the grain-fed group had 2,544 species, whereas the grass-fed group had 1,874 species (Supplementary Figure S1B). This indicates a higher diversity of microbiota in the grain-fed group, in addition to the shared species between the two groups.

**Figure 1:**
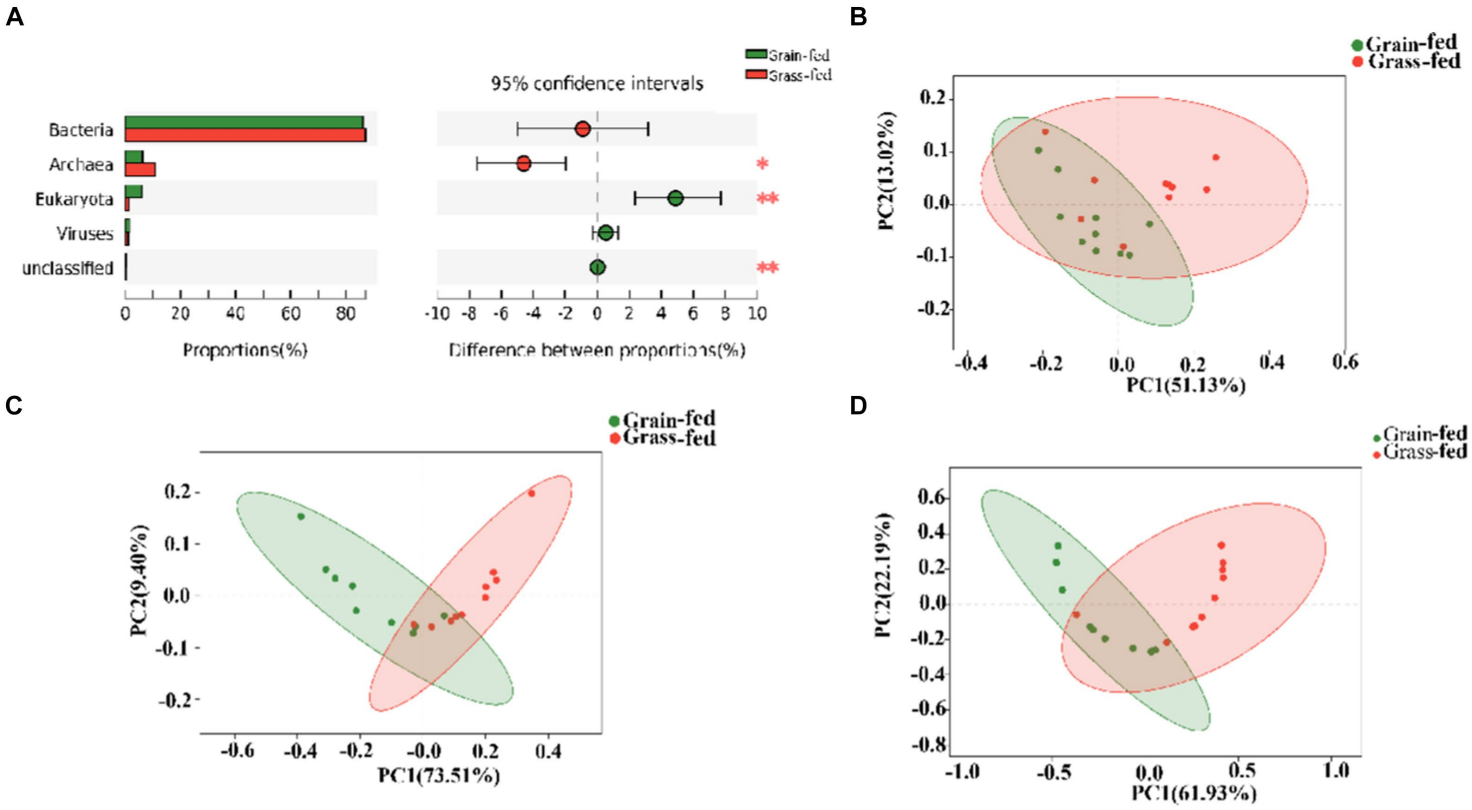
Microbial compositional profiles of grain-fed and grass-fed Sanhe heifers. **(A)** Comparison of microbial domains between grain-fed and grass-fed Sanhe heifers, with significantly different domains tested using the Wilcoxon rank-sum test and an adjusted *p* value of <0.05. ** *p* < 0.01. **(B)** Bacterial compositional profiles of grain-fed and grass-fed Sanhe heifer samples based on species visualized using principal coordinate analysis (PCoA). **(C)** Archaeal compositional profiles of grain-fed and grass-fed Sanhe heifer samples, visualized based on species using PCoA. **(D)** Eukaryota compositional profiles of grain-fed and grass-fed Sanhe heifer samples, visualized on the species level using PCoA.

Figure 1A shows archaea and eukaryotes were found to be significantly different between the two groups (adjusted *p* < 0.01) using the Wilcoxon rank-sum test. PCoA showed separation between the two groups based on bacterial (Figure 1B), archaeal (Figure 1C), and eukaryotic species (Figure 1D). Nevertheless, we focused on comparing bacteria and archaea in the rumen microbiota of grass- and grain-fed Sanhe heifers. Taxon abundance analysis of bacteria revealed 30,255 species belonging to 3,503 genera from 153 phyla, whereas for the archaea taxon, 1,194 species belonging to 245 genera from 23 phyla were identified.

#### 3.2.2 Differences between the rumen microbiota of grass-fed and grain-fed Sanhe heifers

**Figure 2:**
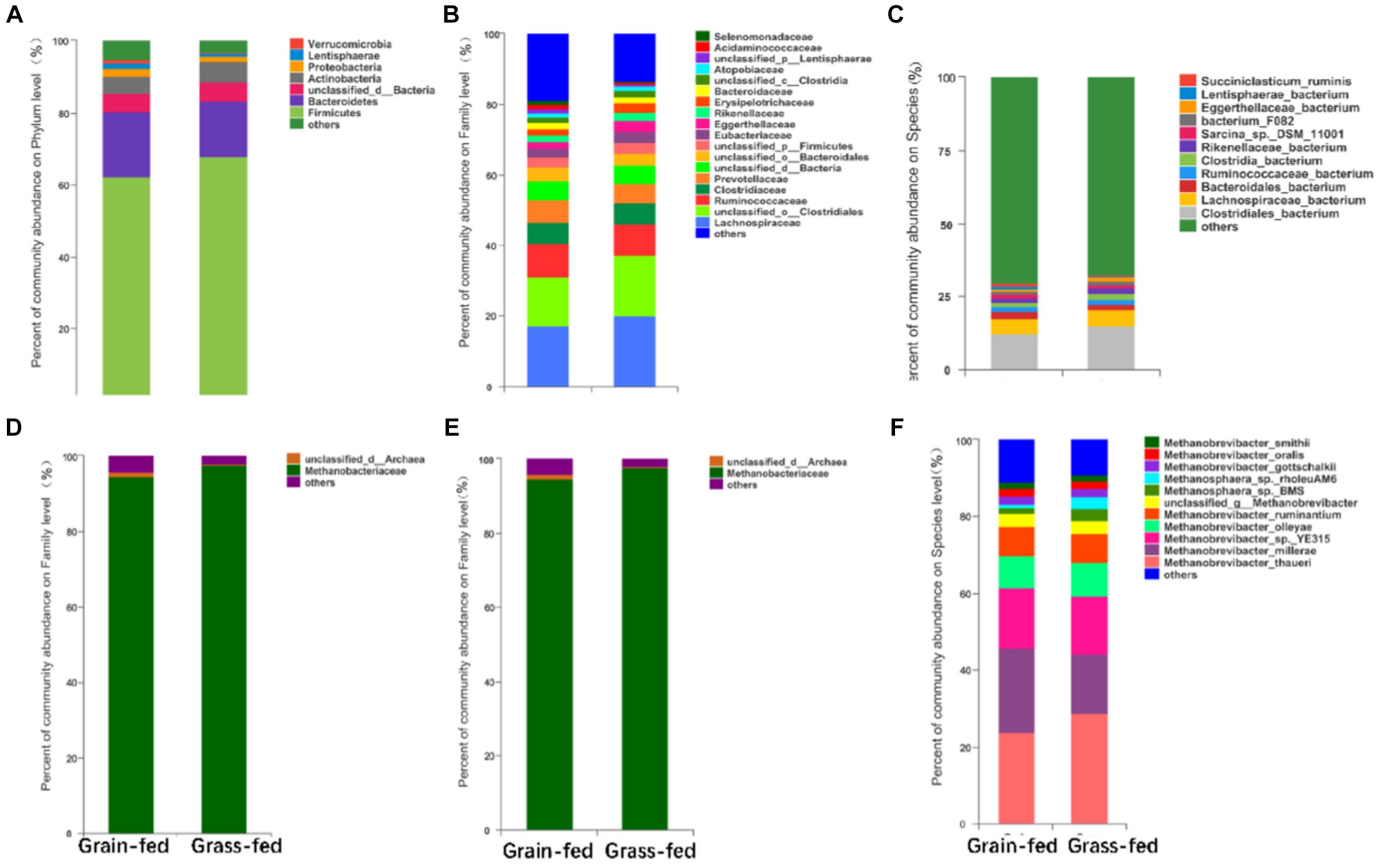
Compositional profiles of the rumen microbiome between grain-fed and grass-fed Sanhe heifers. Relative abundance of **(A)** phyla, **(B)** family, and **(C)** genus of bacterial species; Relative abundance of **(D)** phyla, **(E)** family, and **(F)** genus of archaeal species.

The most abundant bacterial phyla in the rumen microbiota were Firmicutes (64.99%), Bacteroidetes (16.91%), Actinobacteria (5.14%), Proteobacteria (1.81%), Lentisphaerae (1.06%), and Verrucomicrobia (0.70%). The dominant bacterial families were Lachnospiraceae (18.62%), Ruminococcaceae (9.19%), Clostridiaceae (6.05%), Prevotellaceae (5.98%), Eubacteriaceae (2.85%), Eggerthellaceae (2.46%), Rikenellaceae (2.12%), Erysipelotrichaceae (2.10%), Bacteroidaceae (1.79%), Atopobiaceae (1.18%), Acidaminococcaceae (0.81%), and Selenomonadaceae (0.78%, Figure 2A). Prevotella (5.33%), Butyrivibrio (3.43%), Clostridium (3.16%), Ruminococcus (2.66%), Eubacteroides (2.31%), Sarcina (1.58%), Olsenella (1.05%), and Succiniclasticum were the dominant bacterial genera (0.67%, Figure 2B). The dominant bacterial species were Clostridiales_bacterium (13.55%), Lachnospiraceae (5.48%), Bacteroidales_bacterium (2.01%), Ruminococcaceae_bacterium (1.89%), Clostridia_bacterium (1.64%), Rikenellaceae_bacterium (1.52%), Sarcina_sp._DSM_11001 (1.25%), Bacterium_F082 (1.17%), Eggerthellaceae_bacterium (1.04%), Lentisphaerae_bacterium (0.83%), and Succiniclasticum_bacterium (0.67%; Figure 2C).

**Figure 3:**
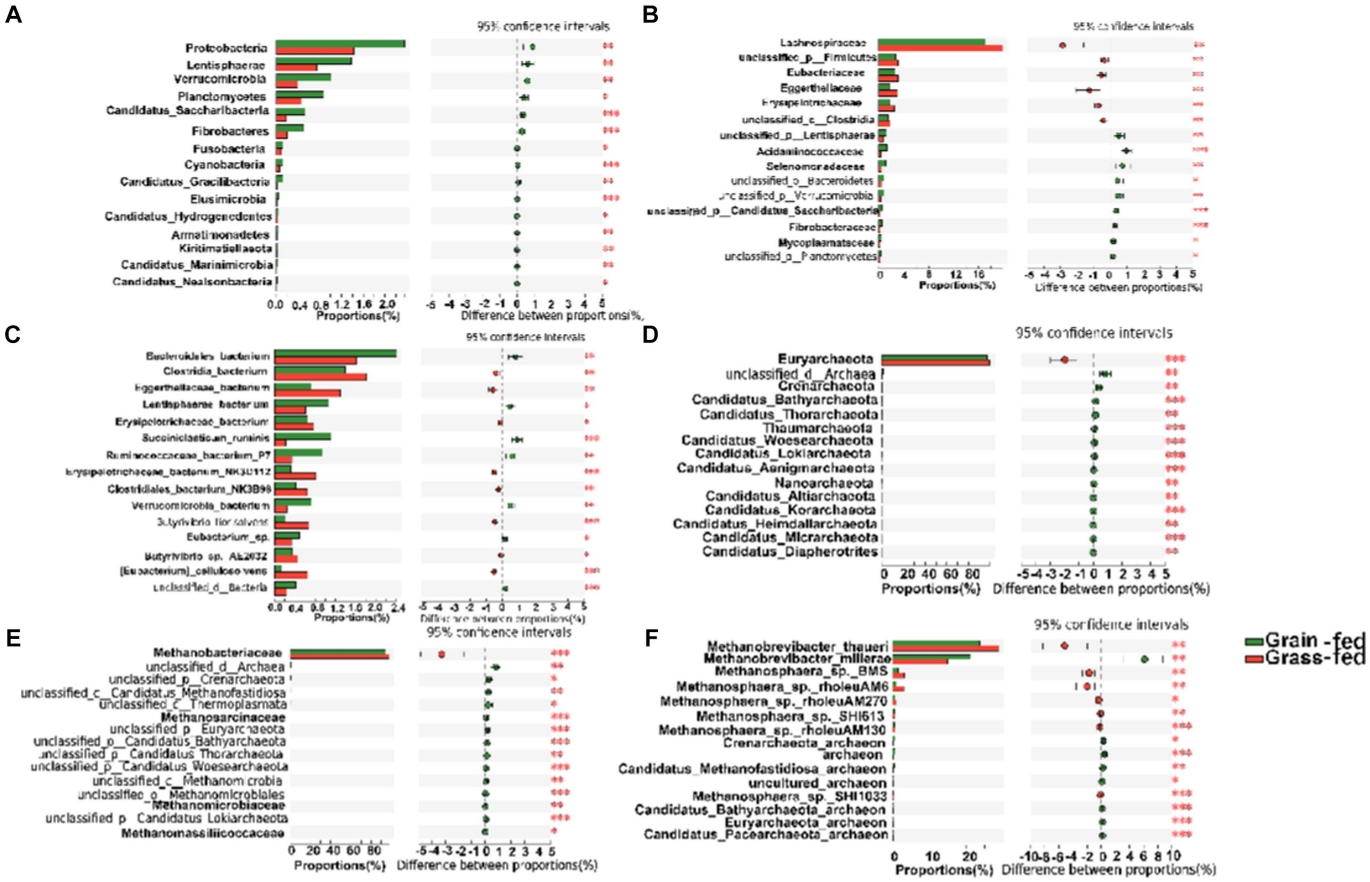
Comparative analysis of the composition of rumen bacteria and archaea between grain-fed and grass-fed Sanhe heifers. **(A)** Comparison of bacterial composition at phylum level. **(B)** Comparison of bacterial composition at family level. **(C)** Comparison of bacterial composition at species level. **(D)** Comparison of archaeal composition at phylum level. **(E)** Comparison of archaeal composition at family level. **(F)** Comparison of archaeal composition at species level. Statistical significance was assessed using the Wilcoxon rank-sum test, with an adjusted *p* value of < 0.05. ** *p* < 0.01.

The differential abundance comparison analysis of bacteria at the phylum level revealed that the abundances of Proteobacteria, Lentisphaerae, Verrucomicrobia, Planctomycetes, Candidatus Saccharibacteria, Fibrobacteres, Fusobacteria, Cyanobacteria, Candidatus Gracilibacteria, Elusimicrobia, Candidatus Hydrogenedentes, Armatimonadetes, Kiritimatiellaeota, Candidatus Marinimicrobia, and Candidatus Nealsonbacteria were significantly higher in the rumen of the grain-fed group than those in the rumen of the grass-fed group (adjusted *p* < 0.05; Figure 3A). At the family level, Lachnospiraceae, Eubacteriaceae, and Eggerthellaceae were highly abundant in the grass-fed group, while the abundance of Acidaminococcaceae, Selenomonadaceae, Fibrobacteraceae, and Mycoplasmataceae were high in the grain-fed group (adjusted *p* < 0.05; Figure 3B). At the species level, Clostridia_bacterium, Eggerthellaceae_bacterium, Erysipelotrichaceae_bacterium, Erysipelotrichaceae_bacterium_NK3D112, Clostridiales_bacterium_NK3B98, Butyrivibrio_fibrisolvens, Butyrivibrio_sp._AE2032, and [Eubacterium]_cellulosolvens were significantly enriched in the grass-fed group. In contrast, Bacteroidales_bacterium, Lentisphaerae_bacterium, Succiniclasticum_ruminis, Ruminocoaceae_bacterium_P7, Verrucomicrobia_bacterium, and Eubacterium_sp. showed significant enrichment in the grain-fed Sanhe heifers (*p* < 0.05; Figure 3C).

The differential analysis of the abundance of archaea in the rumen of heifers in two feeding regimens identified Euryarchaeota (98.13%) as the most abundant archaeal phylum, which was significantly higher in the rumen of grass-fed heifers than in that of grain-fed heifers (adjusted *p* < 0.05; Figures 2D, 3D). At the family level, the abundance of Methanobacteriaceae (95.87%) was significantly enhanced in the grass-fed Sanhe heifers, whereas the abundances of other differential families were all significantly high in the rumen of the grain-fed groups (adjusted *p* < 0.05; Figures 2E, 3E). At the species level (relative abundance >1%), the abundances of Methanobrevibacter_thauer (26.18%), the most abundant archaeal species, followed by Methanosphaera_sp._BMS (2.31%) and Methano sphaera_sp._rholeuAM6 (1.98%) were significantly higher in the rumen of the grass-fed group than those in the rumen of the grain-fed group. In contrast, Methanobrevibacter_millerae (17.99%) was highly abundant in the grain-fed Sanhe heifers (adjusted *p* < 0.05, Figures 2F, 3F).

#### 3.2.3 Different functions of the rumen microbiota between grass-fed and grain-fed groups of Sanhe heifers

The comparison of the KEGG profiles and genes encoding CAZymes identified 329 endogenous third-level pathways as rumen microbial metabolic pathways (Supplementary Table S2). These pathways belonged to six first-level categories.

**Figure 4:**
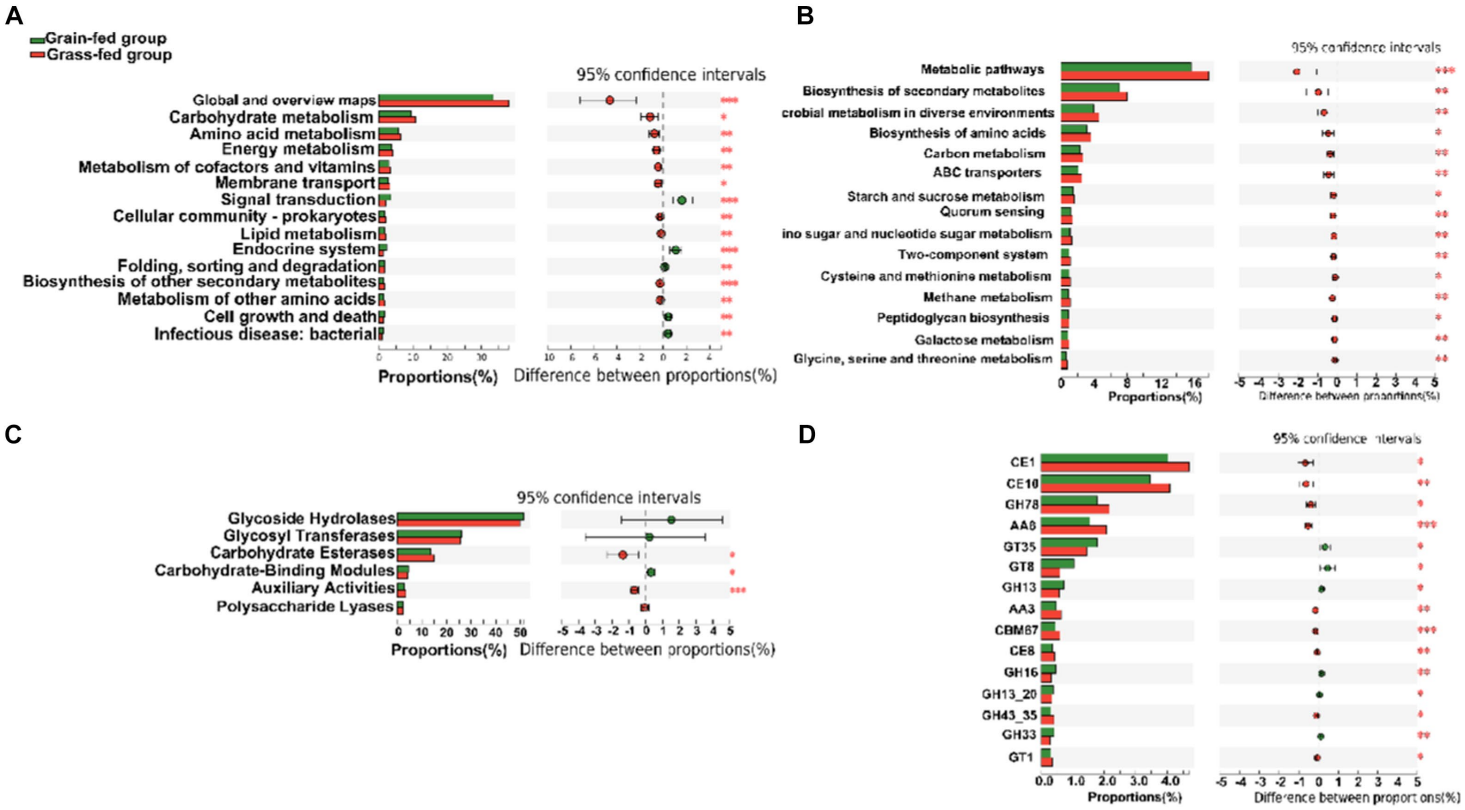
The comparison of KEGG pathways and carbohydrate-active enzymes (CAZy) between grain-fed and grass-fed Sanhe heifers; **(A)** The top 15 KEGG pathways at the second level that were found to be significantly different between the two groups; **(B)** The top 15 of significantly different KEGG pathways at KEGG third-level; **(C)** The carbohydrate-active enzyme (CAZyme) profiles at the class level; **(D)** The top 15 of significantly different CAZyme profiles at the family level.

At the second level of KEGG pathways, 46 functional categories were identified, including “Global and overview maps,” “Carbohydrate metabolism,” “Amino acid metabolism,” “Replication and repair,” “Energy metabolism,” and “Metabolism of cofactors and vitamins.” PCoA indicated a significant separation in the functional potential between the two feed systems (*p* < 0.05; Supplementary Figure S2). Figure 4A displays the top 15 KEGG pathways that were significantly different at the second level. At the third level of KEGG analysis, the top 15 significantly different pathways included “Metabolic pathways,” and “Biosynthesis of secondary metabolites” (Figure 4B). These pathways were enriched in the grass-fed group and belong to three level 1 pathways, including “Environmental Information Processing,” “Genetic Information Processing,” and “Cellular Processes.”

For CAZyme profiles at the family level, 590 genes encoding CAZymes were identified (Supplementary Table S3). Among these, 22 encoded auxiliary activity (AA), 74 encoded carbohydrate-binding modules (CBM), 16 encoded carbohydrate esterases (CE), 259 encoded glycoside hydrolases (GH), 90 encoded glycosyltransferases (GT), and 81 encoded polysaccharide lyases (PL). Six types of CAZymes were identified in the two groups at the class level. Grass-fed Sanhe heifers had an enhanced abundance of GEs and AAs (*p* < 0.05), whereas grain-fed Sanhe heifers exhibited an increased abundance of CBMs (*p* < 0.05, Figure 4C). Furthermore, among the top 15 significantly different CAZyme profiles (Figure 4D), nine were higher in the rumens of the grass-fed group (p < 0.05), including three CE- (CE1, CE10, and CE8), two GH- (GH78 and GH43_35), two AA- (AA6 and AA3), one CBM- (CBM67), and one GT- (GT1) encoding CAZymes. In contrast, six CAZymes encoding GTs (GT35 and GT8) and GHs (GH13, GH16, GH13_20, and GH33) were highly abundant in the grain-fed group (p < 0.05).

#### 3.2.4 Associations between microbial species and microbial functions

**Figure 5:**
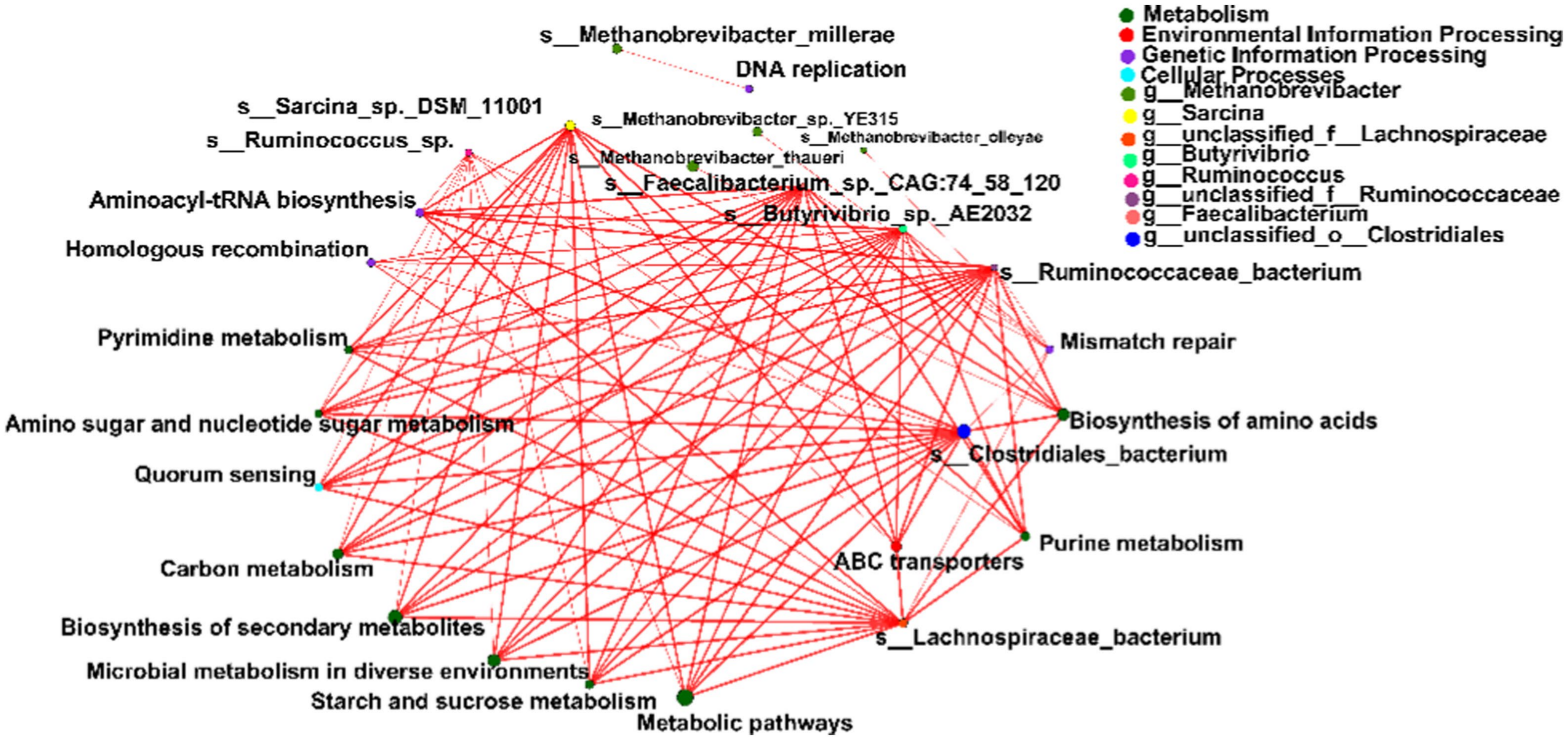
Spearman’s rank correlation constructed between the top 15 differential microbial species and metabolic pathways. Significant differences were determined using the Wilcoxon rank-sum test with an adjusted *p* value of < 0.05. ** *p* < 0.01.

Spearman’s rank correlations were computed to evaluate the relationships between the top 15 differential microbial species and metabolic pathways within the network. Notably, species affiliated with Bacteroidetes, Firmicutes, and Actinobacteria exhibited robust correlations with four primary KEGG pathways: metabolism, environmental information processing, genetic information processing, and cellular processes (|r| > 0.5, *p* < 0.05; Figure 5).

### 3.3 Rumen metabolomic analysis

**Figure 6:**
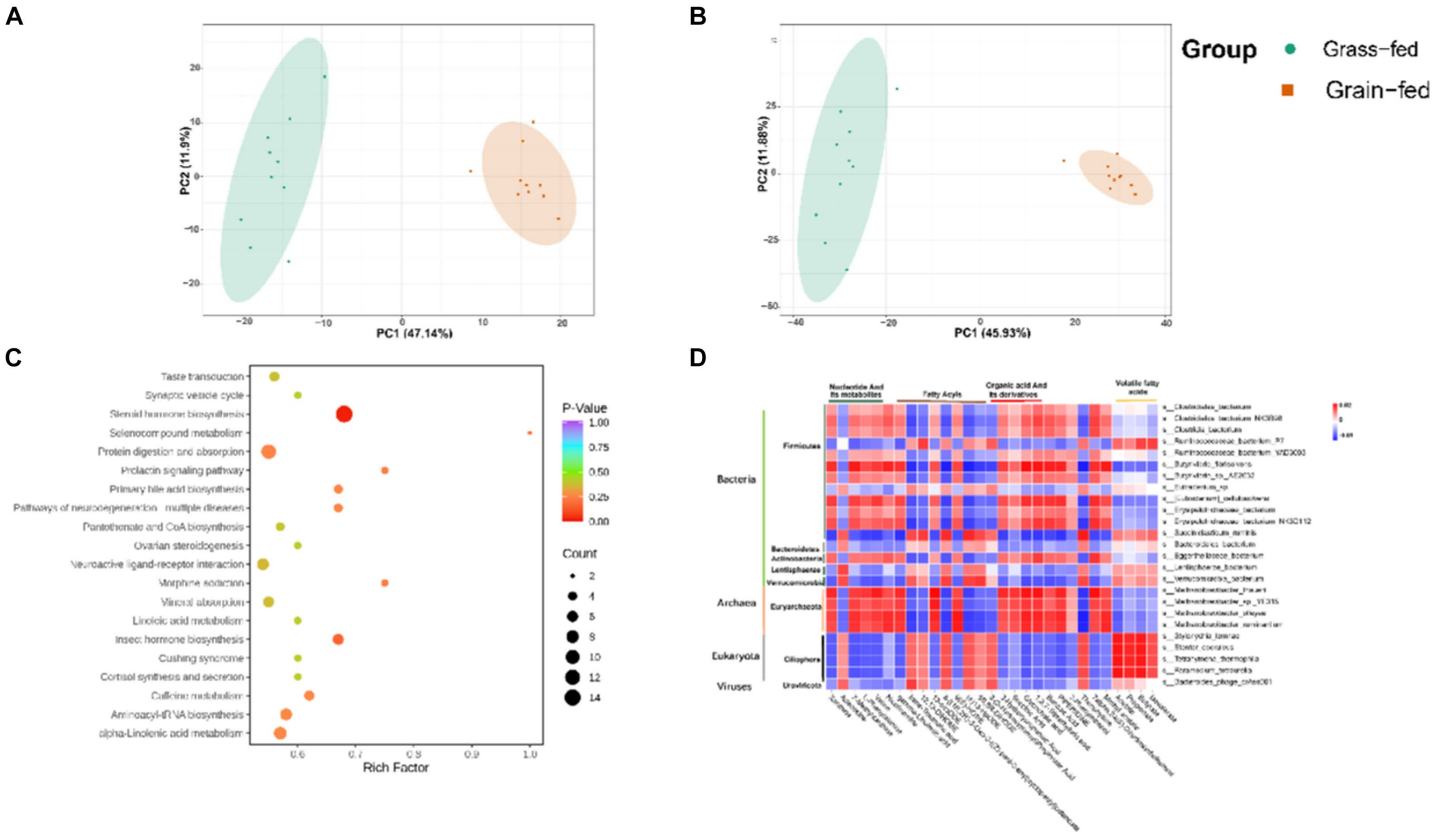
Rumen metabolome comparison of grass-fed and grain-fed Sanhe heifers. **(A)** Scatter plots of the principal component analysis (PCA) model based on all identified metabolite features of rumen samples from the two groups (negative mode); **(B)** Scatter plots of the PCA model based on all identified metabolite features of rumen samples from the two groups (positive mode); **(C)** The pathway enrichment analysis conducted using the significantly different rumen metabolites between grass-fed and grain-fed Sanhe heifers; **(D)** The relationship between rumen fermentation parameters, the top 25 different rumen microbiota, and the top 25 significantly different rumen metabolites using Spearman’s rank correlations.

These rumen metabolites varied significantly between the two groups (Figures 6A,B). Statistical analysis and VIP values from the OPLS-DA analysis supported this finding (Supplementary Figure S3). In total, 2,502 compounds were identified in the ruminal metabolome. After the t-test and VIP filtering for the relative concentrations of rumen metabolites, 1,130 metabolites were significantly different between the two groups of Sanhe heifers (*p* < 0.05, VIP > 1, FC > 2, or FC < 0.5). Among these, 256 metabolites were identified in the negative mode, of which 178 were enriched in the grass-fed group and 78 in the grain-fed group. A total of 874 different metabolites were identified in the positive mode, of which 590 and 284 were enriched in the grass- and grain-fed groups, respectively. Metabolic pathway analysis revealed the enrichment of 20 pathways (Figure 6C), Table 2 displays the differential metabolites identified in the top 20 enriched KEGG pathways. Among these, 68 metabolites were downregulated, and 25 were upregulated.

**Table 2:** Differential metabolites determined using hydrophilic interaction LC–MS analysis of the top 20 enriched differential KEGG pathways between two distinct groups.

Index	Compounds	VIP	*p*_value	Fold_Change	Type
MW0005091	4-Hydroxybenzoic acid	1.354	< 0.001	0.290	Down
MEDP2203	11-Beta-hydroxyandrostenedione	1.422	< 0.001	6.624	Up
MEDN0304	Cinnamic acid	1.229	< 0.001	0.216	Down
MW0105531	Alanine	1.329	< 0.001	0.097	Down
MW0053971	Hydrocortisone	1.086	< 0.001	3.828	Up
MW0109418	Propionic acid	1.359	< 0.001	5.699	Up
MW0006930	Dopamine	1.161	< 0.001	0.183	Down
MW0015246	7alpha,24(S)-Dihydroxycholesterol	1.401	< 0.001	0.010	Down
MW0013923	3alpha,7alpha,12alpha-Trihydroxy-5beta-cholestanate	1.301	0.016	0.052	Down
MW0062186	Progesterone	1.409	< 0.001	0.110	Down
MEDP0160	Adenosine	1.056	0.013	4.554	Up
MEDP1662	NE,NE,NE-Trimethyllysine	1.098	< 0.001	3.856	Up
MW0012191	12,13-DiHOME	1.225	0.001	5.256	Up
MW0141934	2,22-Dideoxy-3-dehydroecdysone	1.183	< 0.001	0.195	Down
MW0169367	L-(−)-Proline	1.256	0.004	0.264	Down
MEDN0149	7-Methylxanthine	1.051	< 0.001	0.424	Down
MEDP1153	20-HETE	1.361	< 0.001	0.058	Down
MEDL00586	L-Arginine	1.303	< 0.001	3.620	Up
MW0144026	8-[(1R,2R)-3-Oxo-2-{(Z)-pent-2-enyl}cyclopentyl]octanoate	1.440	< 0.001	65.459	Up
MW0107942	l-Pipecolic acid	1.392	< 0.001	9.026	Up
MW0143699	5S,8R-DiHODE	1.131	< 0.001	3.133	Up
MW0010776	(+)-Ecdysterone	1.156	< 0.001	0.062	Down
MEDP1136	D-Fructose 6-phosphate	1.244	< 0.001	0.099	Down
MEDN0533	Xanthosine	1.322	< 0.001	0.070	Down
MEDN1424	2,3-dinor-8-iso Prostaglandin F2alpha	1.108	0.001	0.433	Down
MW0102908	trans-Traumatic acid	1.387	< 0.001	12.586	Up
MW0006998	Estrone	1.383	< 0.001	0.114	Down
MW0014686	5alpha-Androstane-3alpha,17beta-diol	1.192	0.003	0.283	Down
MW0052436	DL-2-Methylbutyric acid	1.257	< 0.001	0.131	Down
MW0015783	Androstenediol	1.304	0.003	0.035	Down
MW0109379	Proline	1.259	< 0.001	0.323	Down
MEDP1482	Allopregnanolone	1.272	0.013	0.079	Down
MEDL02347	L-Phenylalanine	1.324	< 0.001	0.165	Down
MEDP0011	L-Lysine	1.379	< 0.001	0.092	Down
MW0015782	Androstanedione	1.406	< 0.001	10.536	Up
MEDP0456	Piperidine	1.335	< 0.001	0.125	Down
MW0013551	2-Methoxyestradiol	1.311	< 0.001	0.042	Down
MW0012540	16alpha-Hydroxyandrostenedione	1.350	< 0.001	0.235	Down
MW0052666	Estrone glucuronide	1.211	< 0.001	3.934	Up
MW0052385	Dihydrotestosterone	1.038	< 0.001	0.397	Down
MEDL00009	L-Valine	1.373	< 0.001	0.074	Down
MW0014734	5beta-Dihydrotestosterone	1.093	< 0.001	7.388	Up
MW0168540	3-(2-Aminoethyl)-1 h-Indol-5-Ol	1.004	< 0.001	0.172	Down
MW0010027	Valine	1.292	< 0.001	0.068	Down
MW0013517	2-Hydroxyestradiol	1.236	< 0.001	6.893	Up
MEDN0208	Melatonin	1.272	< 0.001	0.390	Down
MW0012734	19-Hydroxytestosterone	1.173	< 0.001	0.314	Down
MEDN0778	5,6-EET	1.392	< 0.001	4.637	Up
MEDL00809	Tryptamine	1.174	< 0.001	0.183	Down
MW0008850	N-Arachidonoyl dopamine	1.395	< 0.001	0.023	Down
MW0052998	gamma-Linolenic acid	1.121	< 0.001	0.271	Down
MW0142826	3beta,5beta-Ketodiol	1.417	< 0.001	0.008	Down
MW0010672	Theophylline	1.447	< 0.001	14.710	Up
MW0054379	Linolenic acid	1.424	< 0.001	28.109	Up
MEDP0208	Succinic acid	1.024	0.005	0.294	Down
MEDN1417	(±)13-HpODE	1.438	< 0.001	124.643	Up
MW0142889	3-Dehydroecdysone	1.380	< 0.001	5.044	Up
MW0121074	5-Acetylamino-6-formylamino-3-methyluracil	1.001	< 0.001	4.079	Up
MW0108596	N6,N6,N6-Trimethyl-L-lysine	1.097	0.002	0.261	Down
MEDN0768	13-oxoODE	1.247	< 0.001	0.080	Down
MW0141312	12(13)-EpOME	1.309	< 0.001	0.071	Down
MEDN0140	Xanthine	1.226	< 0.001	0.252	Down
MW0149027	Etherolenic acid	1.332	< 0.001	0.197	Down
MW0015453	9-Hydroperoxy-10E,12Z,15Z-octadecatrienoic acid	1.329	< 0.001	3.140	Up
MW0109196	Phenylalanine	1.289	< 0.001	0.146	Down
MW0009765	Stearidonic acid	1.035	0.004	0.252	Down
MW0141343	12-Oxo-9(Z)-dodecenoic acid	1.093	0.003	0.356	Down

LC–MS, liquid chromatography-mass spectrometry; KEGG, Kyoto Encyclopedia of Genes and Genomes.

### 3.4 Relationship between rumen microbiota, metabolites, and fermentation parameters

Spearman’s rank correlations were determined among rumen fermentation parameters, the top 25 significant rumen metabolites, and the top 25 rumen microbiota. As shown in Figure 6D, 16 bacteria species (*Clostridiales_bacterium*, *Clostridiales_bacterium_NK3B98*, *Clostridia_bacterium*, *Ruminococcaceae_bacterium_P7, Ruminococcaceae_bacterium_YAD3003, Butyrivibrio_fibrisolvens*, *Butyrivibrio_sp._AE2032*, *Eubacterium_sp.*, *[Eubacterium]_cellulosolvens*, *Erysipelotrichaceae_bacterium*, *Erysipelotrichaceae_bacterium*, *Erysipelotrichaceae_bacterium_NK3D112*, *Succiniclasticum_ruminis*, *Bacteroidales_bacterium*, *Eggerthellaceae_bacterium*, *Lentisphaerae_bacterium*, and *Verrucomicrobia_bacterium*); four Archaeon species (*Methanobrevibacter_thaueri*, *Methanobrevibacter_sp._YE315*, *Methanobrevibacter_olleyae*, and *Methanobrevibacter_ruminantium*); four Eukaryota species (*Stylonychia_lemnae*, *Stentor_coeruleus*, *Tetrahymena_thermophila*, and *Paramecium_tetraurelia*); and one virus species (*Bacteroides_phage_crAss001*) were identified to have a strong association with the top 25 different metabolites. These metabolites included five nucleotides and their metabolites, two amino acids and their metabolites, eight fatty acids, five organic acids and their derivatives, one alcohol and amine, one heterocyclic compound, one phenolic acid, one heterocyclic compound, one hormone and its related compounds, and one aldehyde, one ketone, and one ester. The microbiota in this study belonged to five phyla: Firmicutes, Bacteroidetes, Actinobacteria, Lentisphaerae, and Verrucomicrobia. These phyla are primarily associated with the degradation of carbohydrates. Additionally, the microbiota species belonging to Archaea was Euryarchaeota, which is known to be involved in methane production.

## 4 Discussion

Dietary feed type compositions affect not only the available fermentation substrates, but also the ruminal microenvironment, whereas VFA profiles regulate the metabolic pathways of microbiota (Newbold and Ramos-Morales, 2020). In this study, we provide evidence supporting our hypothesis, i.e., that diet-induced changes in the rumen microbiota of Sanhe heifers explain the different effects of two feeding regimes (i.e., grain and grass) on the VFA and metabolite content of the host.

Intensive feeding improves the performance of cattle and provides economic benefits. In this study, grain-fed Sanhe heifers produced more VFA than grass-fed Sanhe heifers, which is consistent with the results of previous studies (Wang et al., 2020). The rumen microbiota plays a crucial role in converting feed into VFAs and provides energy to the ruminants. Consistent with previous studies (Liu et al., 2019), our study demonstrated that rumen volatile acid production in Sanhe heifers differed significantly under different feeding regimes; this differentiation could be attributed to taxonomic and functional differences among the microbes that are supported by the two feeding regimes.

We found a higher diversity of microbiota species in grain-fed Sanhe heifers than in grass-fed Sanhe heifers; however, this result is inconsistent with a previous study (Xu et al., 2021). At the family level, we observed greater abundances of *Lachnospiraceae, Eubacteriaceae, Eggerthellaceae,* and *Erysipelotrichaceae* in the grass-fed group. This finding is expected as the diet exclusively consisted of various grasses, making it conducive to bacterial members associated with fiber degradation. Additionally, it has been suggested that *Lachnospiraceae* strains present in the rumen might be capable of bio-hydrogenating fatty acids (Boeckaert et al., 2009). *Lachnospiraceae* (Vacca et al., 2020) and *Eubacteriaceae* (Zeng et al., 2021) contain butyrate producers. Moreover, *Eggerthellaceae* are involved in the transformation of dietary polyphenols (Selma et al., 2014) and are potential probiotics (Langella et al., 2019). We also showed higher abundances of *Acidaminococcaceae*, *Selenomonadaceae*, *Fibrobacteraceae*, and *Mycoplasmataceae* in the grain-fed group. *Acidaminococcaceae*, an important glutamate-fermenting family of bacteria, produces ammonia as the principal end-product through glutamate fermentation (Zhang L. et al., 2022). *Selenomonadaceae* are common gut inhabitants that are abundant in animals on a high-starch diet and utilize saccharides and lactate (Korpela et al., 2014). *Mycoplasmataceae* are related to the host immune system (Wood et al., 2021). All known methanogens are the members of the Euryarchaeota (Mizrahi et al., 2021). In this study, we revealed that the abundance of *Methanobacteriaceae,* the most prominent family of methanogens, was higher in the grass-fed group than in the grain-fed group. This result explains why grass-fed cattle emit more methane from the perspective of microbial microbiota (Capper, 2012). Methane emissions result in a large loss of energy and decrease animal efficiency (Zhou et al., 2009), which is consistent with our finding that grass-fed Sanhe heifers produce fewer VFAs and more methane than grain-fed Sanhe heifers. However, there are reports that grass-fed systems can decrease carbon footprints (Lynch, 2019).

Metagenomic analyses have been successfully used to determine the composition and role of various microbiome in the rumen (Li and Guan, 2017; Shen et al., 2020). Mu et al. (2022) found that the feeding structure of a high-concentrate diet affected ruminant microbiome composition. We revealed a higher number of KEGG pathways in the grass-fed group compared with the grain-fed group. Most of these pathways are associated with carbohydrate degradation, which may be caused by the different carbohydrate compositions of diets under different feeding systems. Additionally, we found that methane metabolism was higher in the grass-fed group, which is consistent with our previous results of an increased abundance of methane-producing microbiota in the grass-fed group. Regarding the carbohydrate-active enzymes in the rumen microbiome, the GH-encoding genes had the widest distribution in the genomes (Stewart et al., 2018), mainly comprising enzymes involved in polysaccharide metabolism, including cellulose and starch. GTs are enzymes involved in the production of oligosaccharides, polysaccharides, and glycoconjugates and can catalyze the formation of glycosidic bonds to generate glycosides (Breton et al., 2006). Although no significant differences were observed, the abundances of GHs and GTs in the grain-fed group were higher in our study, which is consistent with the results of a previous study (Wang et al., 2019). The abundance of CBMs was higher in the grain-fed group. CBMs are non-enzymatic entities that can improve enzyme catalytic efficiency by preferentially binding to polysaccharides and increasing enzyme concentration (Jones et al., 2018). In contrast, a higher abundance of CEs, AAs, and PLs was identified in the grass-fed group than that in the grain-fed group, which is consistent with the results of Wang et al. (2019). Together, these results indicate that the rumen microbiome of the grain-fed group exhibited a higher prevalence of enzymes such as GHs, GTs, and CBMs, which are involved in starch degradation and binding. This allows them to efficiently digest a relatively larger proportion of concentrate than the grass-fed group. In contrast, a higher abundance of CEs and PLs as cell wall-degrading enzymes than that in the grain-fed group contributed to the digestion of fiber in the grass group.

The metabolomic analyses also revealed the enrichment of pathways related to carbohydrate metabolism, including “Propanoate metabolism,” the grain-fed group supporting our metagenomic results. Moreover, the enriched pathways are associated with lipid metabolism, including “Steroid hormone biosynthesis,” “Primary bile acid biosynthesis,” “Linoleic acid metabolism,” “Arachidonic acid metabolism,” and “Alpha-linolenic acid metabolism.”

In conclusion, we analyzed the dynamics of the rumen microbiota of Sanhe heifers under two different feeding regimes (i.e., grass and grain) using metagenomic and metabolomic analyses. We found that grain intensified rumen fermentation, and that the composition and function of the rumen microbiota varies depending on the feeding system, with notable differences in metabolic pathways, including carbohydrate digestion, amino acid metabolism, and methane production. Other studies employing non-targeted metabolomics have also reported similar outcomes. In summary, omics analysis has revealed noteworthy distinctions in both the composition of the rumen microbiota and their resultant metabolites under different feeding regimes.

## Data availability statement

The original contributions presented in the study are publicly available. This data can be found at: https://www.ncbi.nlm.nih.gov, BioProject: PRJNA942501.

## Ethics statement

The study protocol was approved by the Ethical Committee of China Agricultural University (project number AW22121202-1-2). The studies were conducted in accordance with the local legislation and institutional requirements. Written informed consent was obtained from the owners for the participation of their animals in this study.

## Author contributions

XZ: Writing – original draft. WW: Writing – review & editing. YW: Writing – review & editing. ZC: Writing – review & editing. HY: Writing – review & editing. SL: Writing – review & editing.
