# Supplementary material for: Metagenomic and metabolomic analyses reveal differences in rumen microbiota between grass- and grain-fed Sanhe heifers
Source: Front Microbiol. 2024 May 13;15:1336278. doi: 10.3389/fmicb.2024.1336278 (PMC11128563; doi:10.3389/fmicb.2024.1336278)
Supplement: Supplementary file 1 [file Table_1.DOCX]

**Table S1 Summary of sequence data generated from rumen samples of Grass- and Grain-fed Sanhe heifers.**

| Sample | Raw reads | Raw base（bp） | Clean reads | Clean base(bp) | Contigs | Contigs bases(bp) | N50(bp) | ORFs |
| --- | --- | --- | --- | --- | --- | --- | --- | --- |
| Grain_1 | 114505334 | 17290305434 | 112571158 | 16905842993 | 1709878 | 1025952775 | 611 | 2153795 |
| Grain_2 | 109110806 | 16475731706 | 107112340 | 16121643767 | 1542987 | 930806480 | 622 | 1900328 |
| Grain_3 | 120857328 | 18249456528 | 118882878 | 17885609470 | 1620556 | 1009022378 | 642 | 2011926 |
| Grain_4 | 113325844 | 17112202444 | 111971074 | 16857990537 | 1496491 | 992455301 | 678 | 2127312 |
| Grain_5 | 123648548 | 18670930748 | 121652062 | 18261927831 | 2183207 | 1246440252 | 563 | 2663766 |
| Grain_6 | 112397946 | 16972089846 | 110954044 | 16698424582 | 1888472 | 1063471088 | 550 | 2387244 |
| Grain_7 | 119266446 | 18009233346 | 117847450 | 17708631418 | 1810129 | 1108544724 | 621 | 2361669 |
| Grain_8 | 108020212 | 16311052012 | 106964276 | 16067754139 | 1615019 | 1003714856 | 628 | 2137229 |
| Grain_9 | 110410574 | 16671996674 | 108784568 | 16366696554 | 1591190 | 1063820110 | 701 | 2150528 |
| Grain_10 | 112857756 | 17041521156 | 111259042 | 16744314295 | 1766122 | 1111307649 | 643 | 2321947 |
| Grass_1 | 152766748 | 23067778948 | 150731880 | 22663446278 | 2363380 | 1565776149 | 692 | 3145362 |
| Grass_2 | 120381130 | 18177550630 | 119125818 | 17745522781 | 1581086 | 929015964 | 575 | 2093788 |
| Grass_3 | 107146494 | 16179120594 | 105970696 | 15876474176 | 1587311 | 985204013 | 624 | 2110331 |
| Grass_4 | 121133982 | 18291231282 | 119611446 | 17905147083 | 1937422 | 1097423526 | 556 | 2427400 |
| Grass_5 | 107354308 | 16210500508 | 105381274 | 15852461966 | 1716479 | 1038050847 | 620 | 2142623 |
| Grass_6 | 123088032 | 18586292832 | 121777598 | 18336290790 | 2345372 | 1414806865 | 608 | 2946891 |
| Grass_7 | 112874888 | 17044108088 | 111185610 | 16594389700 | 1576872 | 902880637 | 550 | 2129514 |
| Grass_8 | 129611610 | 19571353110 | 128494624 | 19283831144 | 2282326 | 1355738241 | 588 | 2959719 |
| Grass_9 | 130652544 | 19728534144 | 129337658 | 19423050140 | 2212670 | 1331790820 | 597 | 2907834 |
| Grass_10 | 100503014 | 15175955114 | 99597894 | 14960739745 | 1740374 | 912434975 | 505 | 2157808 |
| Total | 2349913544 | 354836945144 | 2319213390 | 3.4826E+11 | 36567343 | 22088657650 | 12174 | 47237014 |
| Mean | 117495677 | 17741847257 | 115960670 | 17413009469 | 1828367 | 2103681681 | 609 | 2361851 |
| SEM | 2551187 | 385229192 | 2531029 | 380163100 | 65016 | 41122203 | 11 | 81387 |
|  |  |  |  |  |  |  |  |  |


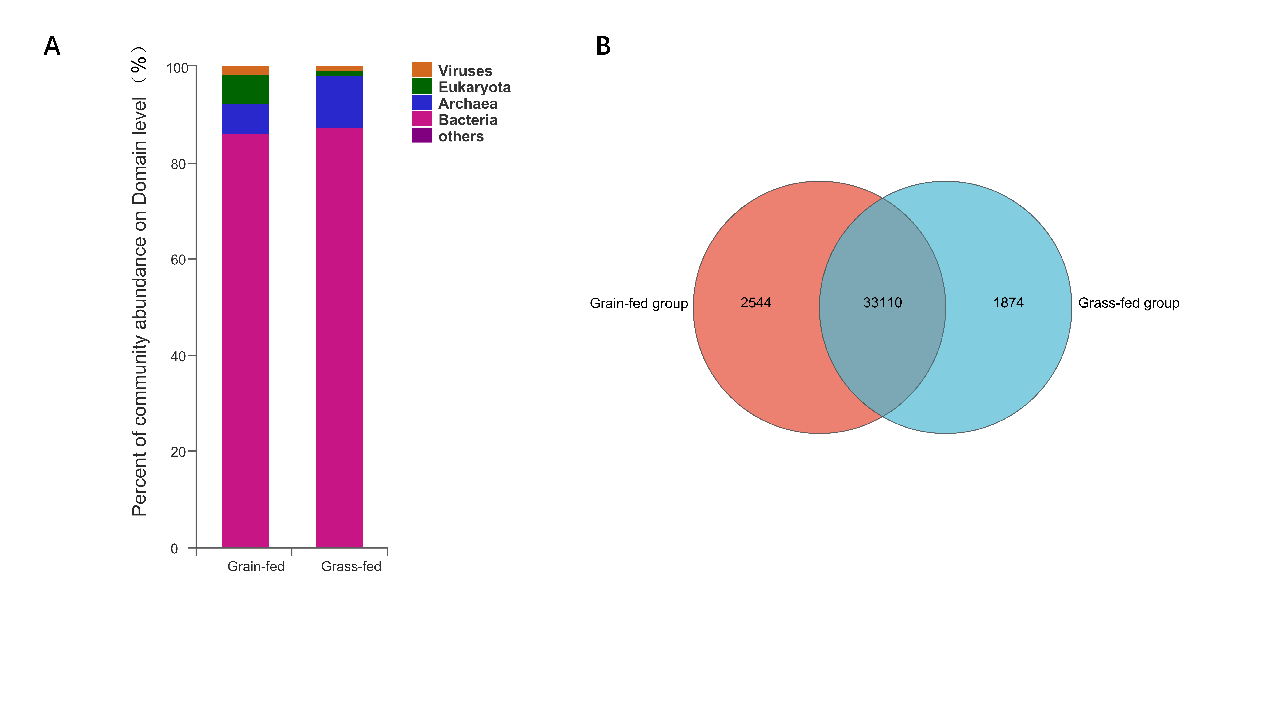


**Figure S1. Profiles of rumen microbial composition of Sanhe heifers. a. The rumen microbial composition based on the domain level taxonomy; b.** **Venn diagram plot of grass-fed and grain-fed Sanhe heifer samples at species level.**

**
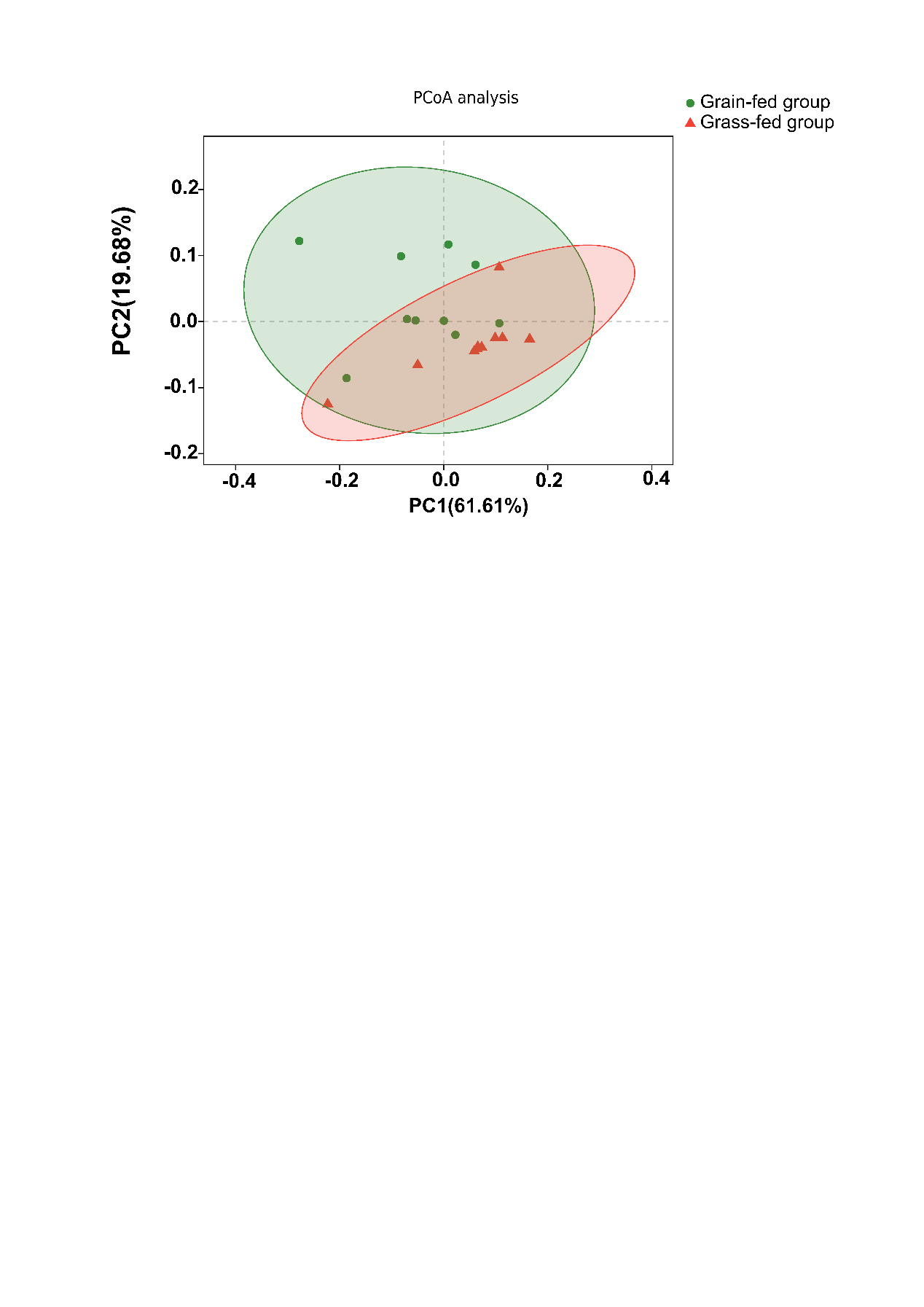
**

**Figure S2. The PCoA indicated a significant separation in KEGG functional potential between the two feed system Sanhe heifers.**

**
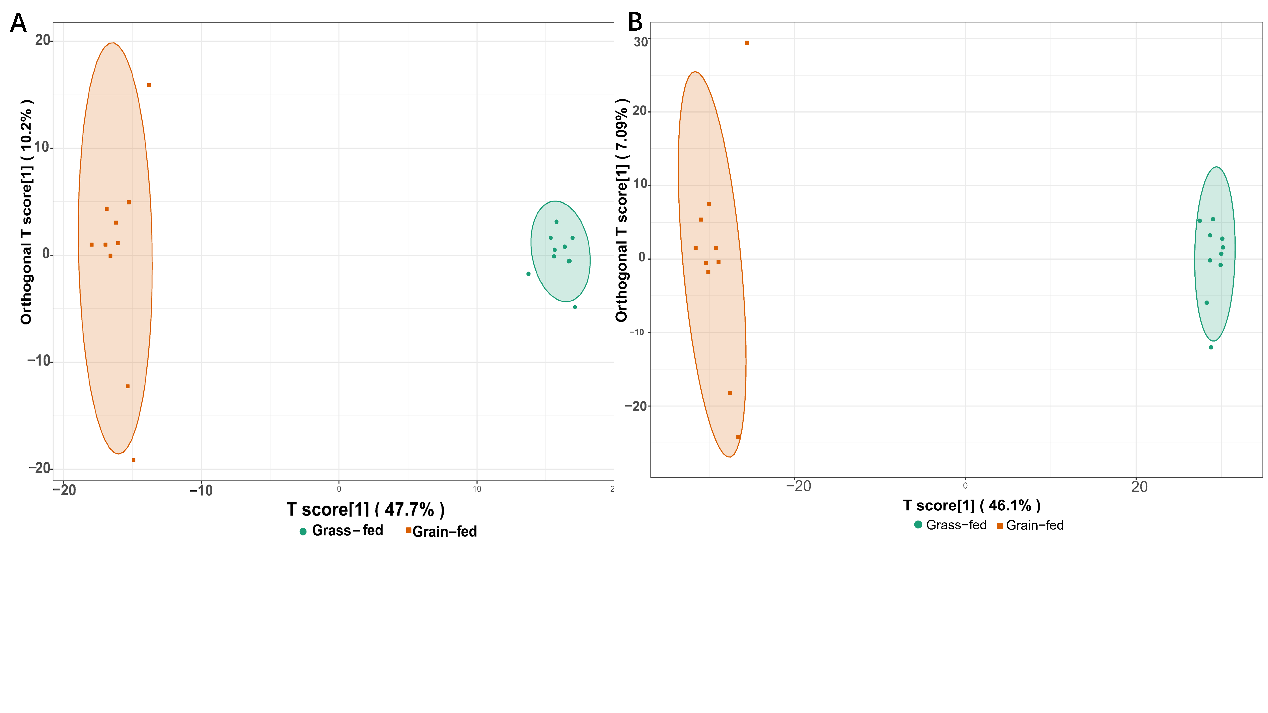
**

**Figure S3. Scatter plots of the Principal component analysis (PCA) model based on all identified metabolite features of rumen samples from the two groups [negative mode(a), positive mode(b)].**
