# Supplementary material for: Metagenomic and metabolomic analyses reveal differences in rumen microbiota between grass- and grain-fed Sanhe heifers
Source: Front Microbiol. 2024 May 13;15:1336278. doi: 10.3389/fmicb.2024.1336278 (PMC11128563; doi:10.3389/fmicb.2024.1336278)
Supplement: Supplementary file 3 [file Table_3.DOCX]

Supplementary Material

# Supplementary Figures and Tables

## Supplementary Figures

**Supplementary Figure S1.** Profiles of rumen microbial composition of Sanhe heifers. a. The rumen microbial composition based on the domain level taxonomy; b. Venn diagram plot of grass-fed and grain-fed Sanhe heifer samples at species level.

**Supplementary Figure S2.** The PCoA indicated a significant separation in KEGG functional potential between the two feed system Sanhe heifers.

**Supplementary Figure S3.** Scatter plots of the Principal component analysis (PCA) model based on all identified metabolite features of rumen samples from the two groups [negative mode(a), positive mode(b)].

## Supplementary Tables

**Supplementary Table S1.** Summary of sequence data generated from rumen samples of Grass- and Grain-fed Sanhe heifers.

**Supplementary Table S2.** Composition of metabolic pathways based on the third-level function in the KEGG between two groups.

**Supplementary Table S3.** Composition of CAZymes based on the class-level and family-level enzymes between two groups.
